# Integrated Analysis of Weighted Gene Coexpression Network Analysis Identifying Six Genes as Novel Biomarkers for Alzheimer's Disease

**DOI:** 10.1155/2021/9918498

**Published:** 2021-07-26

**Authors:** Tingting Zhang, Nanyang Liu, Wei Wei, Zhen Zhang, Hao Li

**Affiliations:** ^1^College of First Clinical Medicine, Shandong University of Traditional Chinese Medicine, Jinan, Shandong Province, China; ^2^Department of Geratology, Xiyuan Hospital, China Academy of Chinese Medical Science, Beijing, China

## Abstract

**Background:**

Alzheimer's disease (AD) is a chronic progressive neurodegenerative disease; however, there are no comprehensive therapeutic interventions. Therefore, this study is aimed at identifying novel molecular targets that may improve the diagnosis and treatment of patients with AD.

**Methods:**

In our study, GSE5281 microarray dataset from the GEO database was collected and screened for differential expression analysis. Genes with a *P* value of <0.05 and ∣log2FoldChange | >0.5 were considered differentially expressed genes (DEGs). We further profiled and identified AD-related coexpression genes using weighted gene coexpression network analysis (WGCNA). Functional enrichment analysis was performed to determine the characteristics and pathways of the key modules. We constructed an AD-related model based on hub genes by logistic regression and least absolute shrinkage and selection operator (LASSO) analyses, which was also verified by the receiver operating characteristic (ROC) curve.

**Results:**

In total, 4674 DEGs were identified. Nine distinct coexpression modules were identified via WGCNA; among these modules, the blue module showed the highest positive correlation with AD (*r* = 0.64, *P* = 3*e* − 20), and it was visualized by establishing a protein–protein interaction network. Moreover, this module was particularly enriched in “pathways of neurodegeneration—multiple diseases,” “Alzheimer disease,” “oxidative phosphorylation,” and “proteasome.” Sixteen genes were identified as hub genes and further submitted to a LASSO regression model, and six genes (*EIF3H*, *RAD51C*, *FAM162A*, *BLVRA*, *ATP6V1H*, and *BRAF*) were identified based on the model index. Additionally, we assessed the accuracy of the LASSO model by plotting an ROC curve (AUC = 0.940).

**Conclusions:**

Using the WGCNA and LASSO models, our findings provide a better understanding of the role of biomarkers *EIF3H*, *RAD51C*, *FAM162A*, *BLVRA*, *ATP6V1H*, and *BRAF* and provide a basis for further studies on AD progression.

## 1. Introduction

Alzheimer's disease (AD), a chronic and progressive neurodegenerative disease, is one of the leading causes of dementia worldwide, accounting for an estimated prevalence of 60%–80% of all cases [1]. Owing to the increase in aging population, AD has become an enormous health burden for families and societies, and the number of AD cases is predicted to increase to 152 million by 2050 [2]. Clinically, AD has multiple complex manifestations, which are characterised by symptoms such as a gradual decline in memory and cognitive impairment as well as defects in judgement, abstraction, language, and attention [3]. Importantly, the main pathological features of AD include the deposition of extracellular *β*-amyloid plaques composed of myloid-*β* peptides, the formation of intracellular neurofibrillary tangles, and the loss and damage of neurons [4]. Currently, multiple pharmacological treatments, such as donepezil, galantamine, and rivastigmine [5, 6] as well as memantine [7], have been employed to provide temporary relief from symptoms. Although substantial efforts have been made to study the pathology and underlying pathogenesis of AD, there are still no comprehensive therapeutic interventions for AD. Therefore, there is an urgent need to identify novel molecular targets that can improve the diagnosis and treatment of patients with AD.

With the development of technology involving high-throughput sequencing and microarray, bioinformatics is increasingly being used to analyse genetic changes in tumours and nervous systems, thereby providing novel intervention targets and novel therapeutic methods for diseases. The Gene Expression Omnibus (GEO) database is a publicly available genomic repository containing gene expression profiles and the corresponding clinical traits of multiple diseases. Weighted gene coexpression network analysis (WGCNA) is a powerful screening tool to explore the relationship between genes with similar expression patterns and external clinical information by constructing free-scale gene coexpression networks [8] and has been widely verified in multiple diseases [9–16]. Consequently, hub genes highly associated with clinical features have been identified as potential biomarkers and therapeutic targets. This type of systems biology algorithm, to a certain extent, overcomes the limitation that a majority of studies only focus on the expression of differential genes and neglect the high correlation of genes.

**Figure 1:**
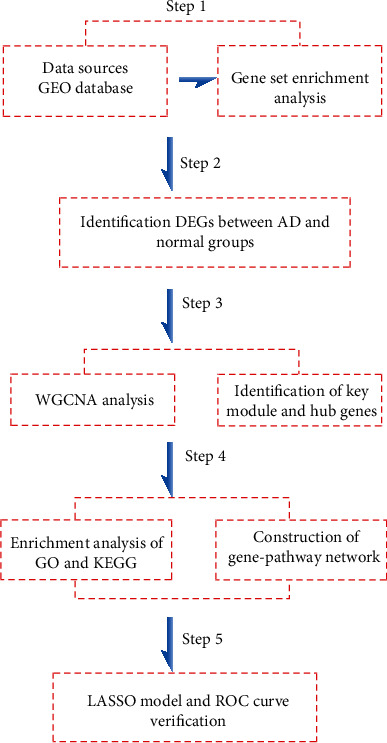
Workflow to identify the key module and hub genes of Alzheimer's disease.

In this study, the public AD dataset GSE5281 from the GEO database was collected for systemic analyses. First, using a combined approach of differentially expressed gene (DEG) analysis and clinical trait-based WGCNA, we further profiled and identified a panel of AD-related coexpression genes by comparing control and AD patients. Functional analyses were performed to determine the characteristics and functions of these genes. Subsequently, we established an AD-related 6-mRNA prediction model using logistic regression and least absolute shrinkage and selection operator (LASSO) analyses. This model was verified using a receiver operating characteristic (ROC) curve. Therefore, these findings will aid in further understanding the underlying mechanisms of AD and highlight the potential application of these targets in AD treatment strategies. The detailed workflow is shown in Figure 1.

## 2. Materials and Methods

### 2.1. Data Mining

The GEO database includes high-throughput gene expression data submitted by researchers worldwide. Large sample sizes were considered to provide more reliable results in the screening of DEGs. Therefore, this research employed a gene expression profile dataset of AD that was downloaded from the GEO public database (http://www.ncbi.nlm. https://nih.gov/geo). The GSE5281 dataset containing 74 control samples and 87 AD samples based on the GPL570 platform was selected for further analysis in the present study. Data from the GEO database are accessible and free of charge, and their utilization does not require the approval of an Ethics Committee.

### 2.2. Gene Set Enrichment Analysis (GSEA)

GSEA is an algorithm based on gene sets that is used to construct a database of molecular characteristics in accordance with known information, including gene characteristics, location, and biological functions [17]. This computational method was used to screen and analyse biological process (BP), cellular component (CC), molecular function (MF), and Kyoto Encyclopaedia of Genes and Genomes (KEGG) pathways that may be associated with AD in the GSE5281 dataset using GSEA v4.1.0 (http://http://www.broad.mit.edu/gsea/). Based on default parameters, GSEA was performed using c5.bp. v6.2.symbols.gmt, c5.go.cc.v7.2.symbols.gmt, c5.go. mf. v7.2.symbols.gmt, and c2.cp. kegg. v6.2.symbols.gmt datasets in the MsigDB V6.2 database [18] as reference gene sets. *P* < 0.05 and false discovery rate (FDR) value < 0.25 were used as cut-off values.

### 2.3. Identification of DEGs

We first converted the probes into gene symbols for a series matrix file of the gene dataset for further analysis. The processed data were subjected to normalisation and log base 2 transformation using the GSE5281 dataset, which was screened for differential expression analysis between AD and healthy tissues, which was performed using the “Limma” package in R software version 3.6.0. Genes with *P* value <0.05 and ∣log2FoldChange | >0.5 were considered DEGs. A heat map cluster and volcano plot of the DEGs were created using the “pheatmap” and “ggplots” packages via R software.

### 2.4. Construction of Weighted Gene Coexpression Network

To explore the expression and interactions of DEGs in AD samples, a gene coexpression network was constructed by the “WGCNA” package in R software [8] according to the following process. We applied the Fragments Per Kilobase Million (FPKM) method to standardise the DEGs of the data matrix and remove the nonstandard data (if the mean FPKM was greater than 0.5, it was defined as standard data). After removing the abnormal samples based on cluster trees, we calculated the Pearson correlation coefficient cor (*i*, *j*) to determine the correlation between gene pairs. The formula fora similar expression matrix is as follows:

(1) $$\begin{array}{c} {a_{i}}_{j}={\left(0.5\times \left(1+\text{cor}\left(i,j\right)\right.\right)}^{\beta }, \end{array}$$

where *a*_*ij*_ is the adjacent function between genes *i* and *j*. To ensure a scale-free network, a soft thresholding power *β* value of 5 was chosen, and the similarity matrix was converted into an adjacency matrix. Subsequently, we built a topological overlap matrix (TOM) to measure the mean network connectivity of each gene. Genes with similar expression profiles were classified into different modules using the dynamic tree cut method based on the relevant parameters (deepSplit of 2 and minModuleSize of 30), and the cutHeight value was set to 0.9. A tree diagram was then built by hierarchical clustering to calculate the correlation between module eigengenes (MEs) and traits, which were used to screen the MEs. The module with the highest correlation with AD among all modules was identified as the most critical module for further analysis and visualised using Cytoscape (version 3.7.2) software. The hub genes in the critical module referred to those that met the following criteria: gene significance (GS) > 0.6 and module membership (MM) > 0.8.

### 2.5. Functional Enrichment Analysis of the Key Module

To further clarify the potential biological implications of the genes in the key module, we performed Gene Ontology (GO) term and KEGG pathway analyses using the “clusterProfiler” package in R software [19]. The three categories of biological process (BP), molecular function (MF), and cellular component (CC) constituted the GO term. In addition, only when the GO or KEGG terms exhibited an FDR of less than 0.05, they were considered significant. Thus, using either BP, CC, or MF analysis as a baseline, the top five terms were selected and further visualised using the GOplot package in R software version 3.6.0. [20] However, KEGG enrichment analysis result was presented visually using a bubble plot. In addition, a gene-KEGG pathway network was established using Cytoscape software (version 3.7.2).

### 2.6. Establishment of a LASSO Model and ROC Curve Analysis

A LASSO model was established to identify the best features for high-dimensional data owing to its strong predictive value and low correlation [21, 22]. The “glmnet” package in R software was used to establish the LASSO model based on the gene expression profiles of hub genes, which could strongly distinguish between AD and control. The minimum lambda value was then used as a reference to identify the best variable to be included in the model. Genes acquired from the LASSO model were used to perform logistic regression analysis for calculating the expression value and regression coefficient of hub genes according to the following formula:

(2) $$\begin{array}{c} index=ExpGene1\times Coef1+ExpGene2\times Coef2+ExpGene3\times Coef3+\cdots +ExpGeneN\times CoefN, \end{array}$$

where “Exp” refers to the expression value of a gene and “Coef” refers to the regression coefficient of a gene. Additionally, ROC curve analysis was employed to evaluate the stability and sensitivity of the LASSO model in identifying AD, which was realised using the pROC package in R software version 3.6.0 [23].

## 3. Results

### 3.1. Functional Enrichment Analysis

**Figure 2:**
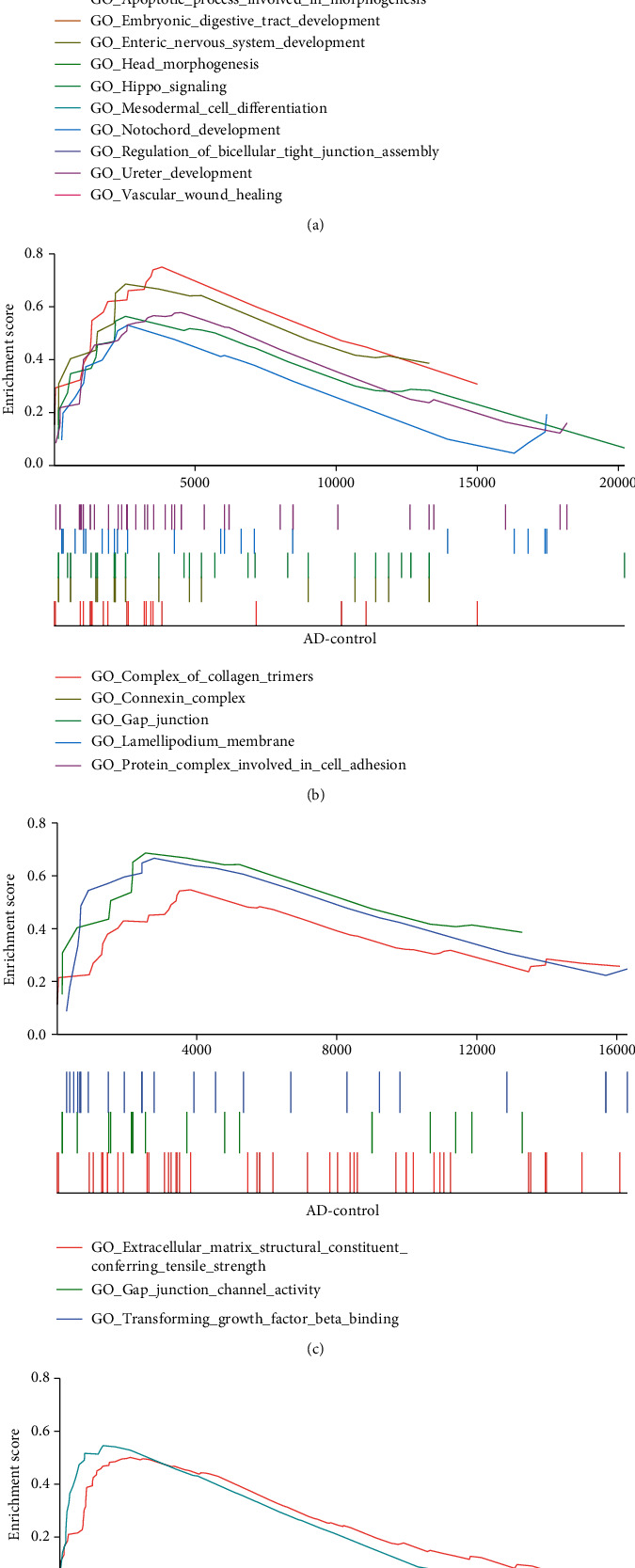
Results of gene set enrichment analysis in AD. (a) Biological processes, (b) cellular component, (c) molecular function, and (d) KEGG pathway.

GSEA was performed using data from the AD and control groups, and the results are shown in Figure 2. The results indicate that with the BP class mediator as a reference, 53 gene sets were significantly enriched in apoptotic processes involved in morphogenesis, enteric nervous system development, notochord development, mesodermal cell differentiation, and head morphogenesis in the AD samples (Figure 2(a) and Supplementary Table 1). Similarly, compared with the control group, CC terms such as complex of collagen trimers, connexin complex, gap junction, lamellipodium membrane, and protein complex involved in cell adhesion were markedly enriched in the AD group (Figure 2(b)). Moreover, extracellular matrix structural constituents conferring tensile strength, gap junction channel activity, and transforming growth factor *β* binding of MF were mainly enriched in the AD group (Figure 2(c)). Furthermore, we found that the AD group was overrepresented in terms of ECM receptor interaction (NES = 1.71, nom *P* value = 0.01) and the NOTCH signalling pathway (NES = 1.73, nom *P* value = 0.02) (Figure 2(d)).

### 3.2. Identification of DEGs between AD and Control Groups

**Figure 3:**
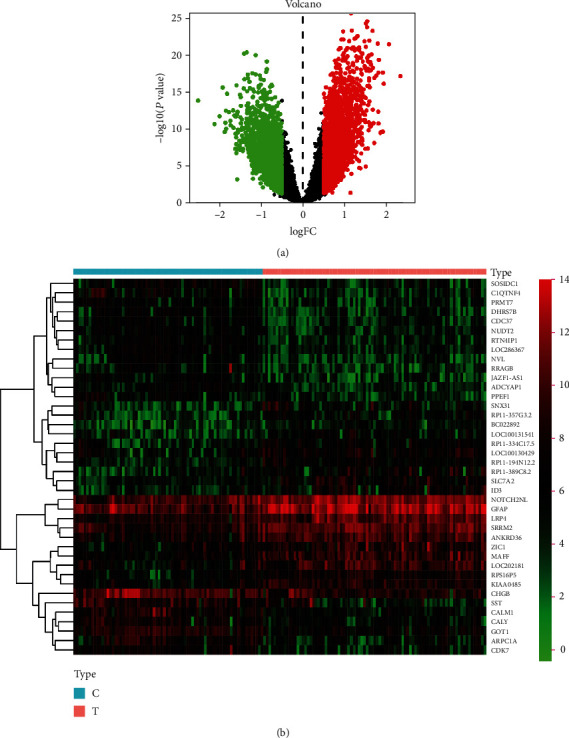
Visualization plots of differentially expressed genes. The red dots represent significantly upregulated genes, and the green dots represent significantly downregulated genes. (a) Volcano plot and (b) a heat map of 40 most differentially expressed genes.

To identify genes that markedly affect AD, we first obtained genes that were differentially expressed between AD patients and controls from the GEO database. We then sorted these genes according to the threshold values of *P* < 0.05 and ∣log2FoldChange | >0.5 and found that among them, 2349 genes were downregulated and 2325 were upregulated. The relevant DEGs are illustrated using a volcano plot (Figure 3(a)). A heat map representing the top 40 genes is presented in Figure 3(b).

### 3.3. WGCNA and Identification of the Key Module and Hub Genes

**Figure 4:**
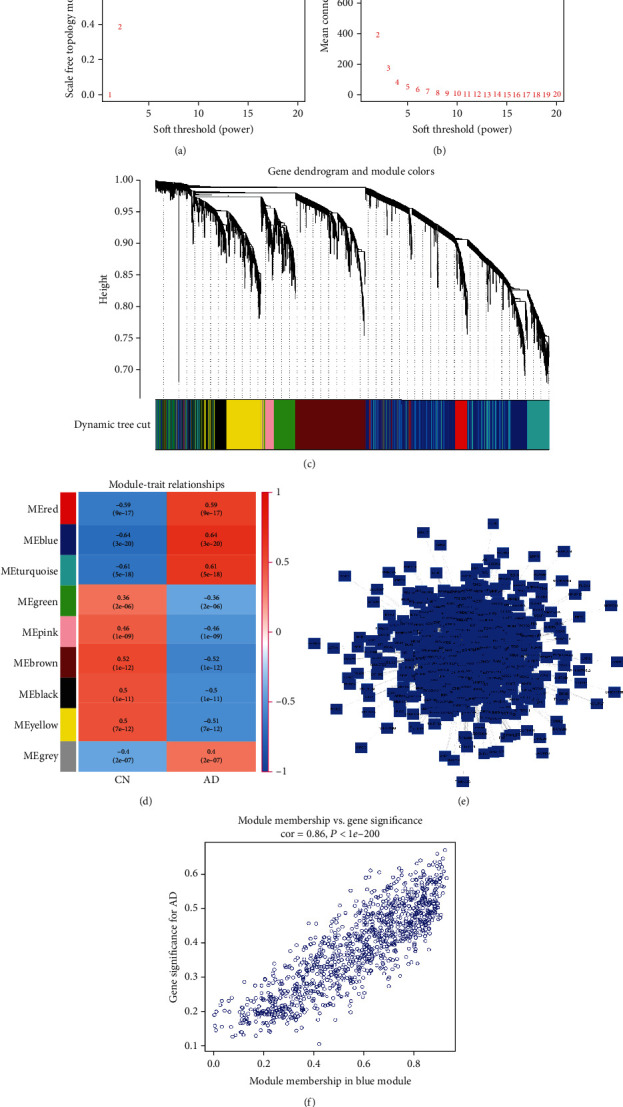
Results of weighted gene coexpression network analysis. (a) Analysis of the scale-free fit index for various soft-thresholding powers (*β*), (b) analysis of the mean connectivity for various soft-thresholding powers, (c) clustering dendrogram, (d) module-trait associations evaluated by correlations between MEs and clinical traits; red represents positive correlation with the clinical trait, and blue represents negative correlation with the clinical trait, (e) protein–protein interaction network of the blue module, and (f) the correlation of module membership and gene significance in the blue module.

After removing the abnormal samples and screening the genes, the expression profiles of 4665 genes including a total of 161 samples in the GEO dataset were extracted for constructing the weighted gene coexpression network using the package “WGCNA” in R software. The key parameter associated with a scale-free network is the soft threshold power value. In the present study, when the soft threshold power was confirmed as five, the scale independence reached 0.9 (Figure 4(a)), and the adjacency matrix gained a comparatively higher mean connectivity value (Figure 4(b)). When merging modules with dissimilarities of less than 10% and minimum modules of less than 30 into larger modules, nine distinct coexpression modules were identified completely via dynamic tree cutting (Figure 4(c)). Furthermore, a correlation analysis between the modules and phenotypes of clinical traits was performed. As shown in Figure 4(d), the blue module showed the highest positive correlation with AD (*r* = 0.64, *P* = 3*e* − 20) and was selected for further analysis. In addition, the brown module showed the highest negative correlation with AD (*r* = −0.52, *P* = 1*e* − 12). Thereafter, a protein–protein interaction network was established based on the genes present in the blue module with a weighted value of greater than 0.2 (Figure 4(e)). Additionally, we performed a correlation analysis between MM and GS for each node in each module. Among them, the correlation value of the blue module was 0.86 (*P* < 1*e* − 200), as depicted in Figure 4(f).When the genes in the blue module met the criteria GS > 0.6 and MM > 0.8, these genes were defined as hub genes and used for further analysis. In total, 16 genes (*ATP5C1*, *PSMD1*, *ATP5B*, *EIF3H*, *EMC4*, *PSMB7*, *RAD51C*, *FAM162A*, *RAP1GDS1*, *BRAF*, *NME1*, *AP3M2*, *RRAGA*, *BLVRA*, *PSMD4*, and *ATP6V1H*) fit all these criteria.

### 3.4. GO and KEGG Pathway Analyses of the Key Module

**Figure 5:**
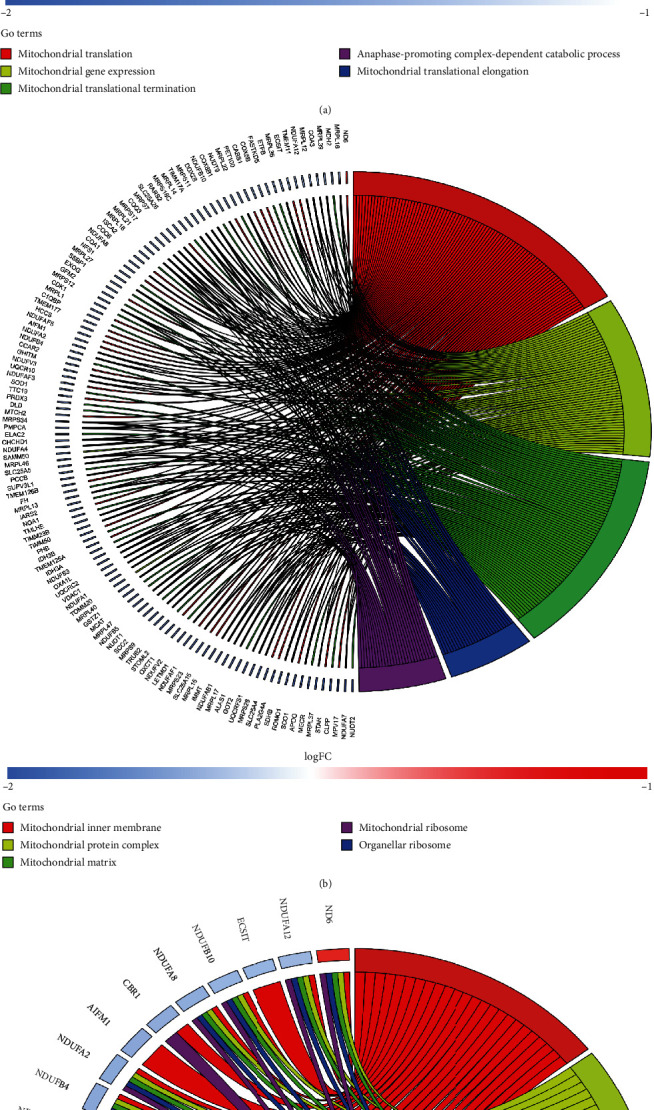
Go analysis of the key module. (a) Biological process; (b) cellular component; (c) molecular function. Notes: chord diagram displays the top five enriched terms.

**Figure 6:**
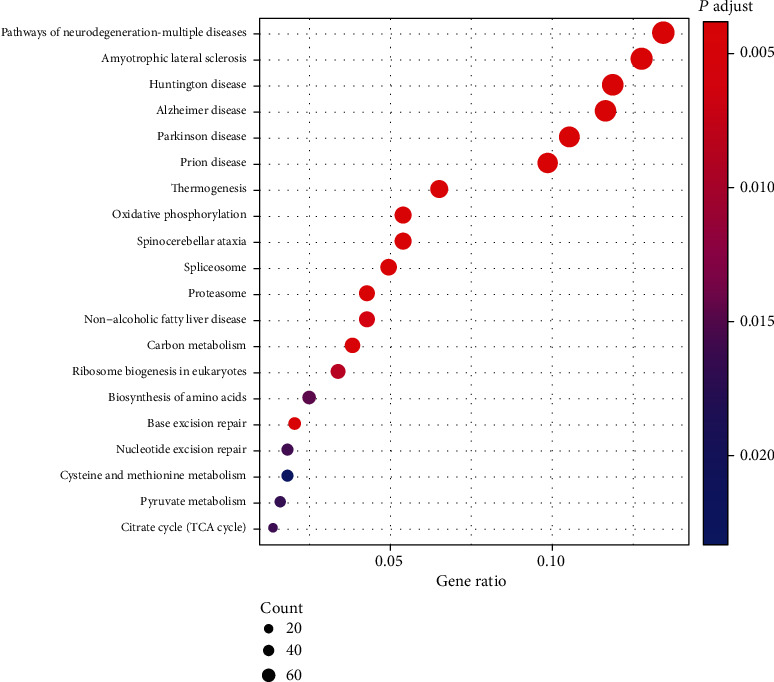
KEGG pathway of the key module. Pathways with *P* < 0.05 were identified as significant. The dot size represents the number of genes, and colour represents the *P* value.

To better interpret the underlying biological roles of these genes in the blue module, we subjected them to GO and KEGG enrichment analysis using the “clusterProfiler” package in R software (Figures 5 and 6). In total, 272 GO-BP, 88 GO-CC, 30 GO-MF, and 22 KEGG pathways were enriched. Regarding BP enrichment, the blue module primarily participated in “mitochondrial translation,” “mitochondrial gene expression,” “mitochondrial translational termination,” “mitochondrial translational elongation,” and so on, a chord diagram of the top five enriched BP terms across gene lists is presented in Figure 5(a) and Supplementary Table 2. The top five terms in CC were mainly enriched in the “mitochondrial inner membrane,” “mitochondrial protein complex,” “mitochondrial matrix,” “organellar ribosome,” and “mitochondrial ribosome” (Figure 5(b) and Supplementary Table 2). The markedly enriched MF terms were “oxidoreductase activity, acting on NAD (P)H,” “NADH dehydrogenase activity,” “NADH dehydrogenase, (ubiquinone) activity,” “NADH dehydrogenase (quinone) activity,” and “oxidoreductase activity, acting on NAD (P) H, quinone or similar compound as acceptor,” as shown in Figure 5(c) and Supplementary Table 2. Furthermore, based on KEGG analysis, the genes in the blue module were particularly enriched in “pathways of neurodegeneration - multiple diseases,” “Alzheimer disease,” “oxidative phosphorylation,” “proteasome,” and other pathways. The visualisation results are presented in Figure 6.

**Figure 7:**
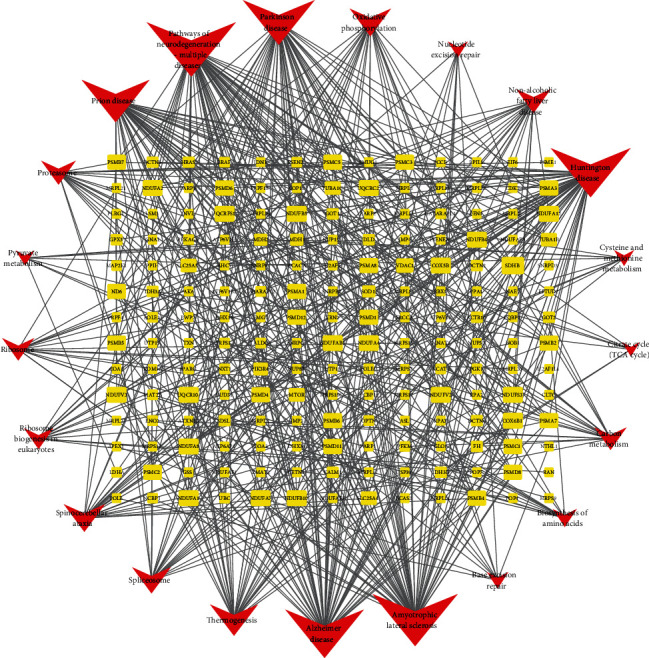
Interaction network of gene pathways. Topological analysis result was calculated using degree. The yellow circles represent the corresponding genes, and the red Vs represent different signal pathways. A larger size represents a larger degree.

In addition, we established a gene-pathway network according to the KEGG signalling pathways and corresponding enrichment genes using Cytoscape, as depicted in Figure 7. This network clearly demonstrated the interaction and crosstalk between multiple signalling pathways and genes.

### 3.5. Establishment of the LASSO Model and Assessment of the ROC Curve

**Figure 8:**
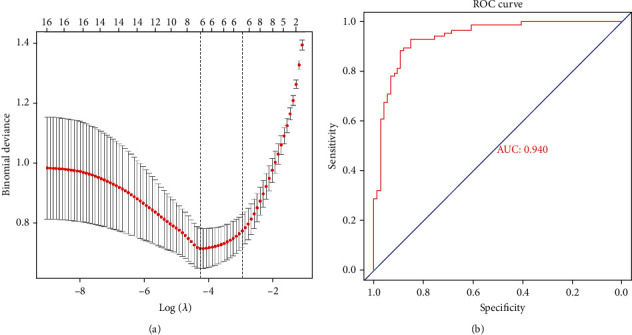
Establishment of a model for predicting AD and its verification. (a) LASSO model. (b) ROC curve analysis of GSE5281.

The expression profile of the selected hub genes was extracted and used to establish the LASSO model (Figure 8(a)). Then, 16 genes were further subjected to a LASSO regression analysis based on the value of lambda.min = 0.0128535, and six genes (*EIF*3*H*, *RAD*51*C*, *FAM*162*A*, *BLVRA*, *ATP*6*V*1*H*, and *BRAF*) were identified to construct the gene signature using nonzero regression coefficients. Furthermore, these six genes were identified based on the model index according to the following formula: index = *EIF*3*H* × (−1.4724261) + *RAD*51*C* × (−0.4871083) + *FAM*162*A* × (−0.3658030) + *BRAF* × (−1.1119874) + *BLVRA* × (−1.1758151) + *ATP*6*V*1*H* × (−0.5092112). Additionally, we assessed the accuracy of the LASSO model by creating an ROC curve, which was designated using the AUC value. As shown in Figure 8(b), the AUC value of the six-gene-based model was 0.940, indicating that these genes may serve as potential biomarkers of AD for further testing.

## 4. Discussion

As an incurable neurodegenerative disease, suitable prevention and treatment methods for AD have been a major but unresolved problem. FDA-approved anti-AD pharmacological therapies, such as donepezil, galantamine, rivastigmine, and memantine, can be used to improve the clinical symptoms of patients with AD; however, these drugs only delay the progression of the disease and do not serve as a curative treatment [5–7]. In addition, AD is accompanied by a huge economic burden. Hence, the exploration of potential mechanisms and therapeutic targets for patients with AD and the construction of a predictive model for assessment are particularly important. In the present study, we focussed on the potential targets of AD and their prognostic values. DEGs between the AD and normal tissues across the GSE5282 dataset were statistically analysed using the WGCNA and LASSO regression methods. The blue module was significantly positively correlated with AD was selected for further analysis, and in this model, six genes (*EIF3H*, *RAD51C*, *FAM162A*, *BLVRA*, *ATP6V1H*, and *BRAF*) were identified as hub genes.

Additionally, we explored the biological processes and signalling pathways associated with AD. According to the results of GSEA, the Notch signalling pathway and ECM receptor interaction were enriched in the AD group. GO enrichment analysis demonstrated that the module with a strong positive correlation with AD participated in biological processes associated with mitochondria, including mitochondrial translation, mitochondrial gene expression, mitochondrial inner membrane, mitochondrial protein complex, NADH dehydrogenase activity, and oxidoreductase activity, acting on NAD (P)H.Mitochondrial dysfunction and oxidative stress may contribute to promoting the accumulation of amyloid-*β* peptides (A*β*) and enhancing the phosphorylation levels of Tau [24]. Moreover, the results of KEGG pathway analysis mainly highlighted categories such as “pathways of neurodegeneration-multiple diseases,” “Alzheimer disease,” “oxidative phosphorylation,” and “proteasome.” For example, the proteasome and its downstream effects have a dual effect on AD symptoms [25]. Proteasomes can degrade A*β*, thereby improving AD [26, 27].

An important finding of this study was that the downregulation of *EIF3H*, *RAD51C*, *FAM162A*, *BLVRA*, *ATP6V1H*, and *BRAF* was closely related to the occurrence of AD.EIF3H, a subunit of the eukaryotic translation initiation factor 3 complex, regulates protein translation, and plays a key role in several processes in the initiation of protein synthesis [28]. It has been rarely reported in AD, but its amplification and overexpression have been noted in multiple tumour tissues, including colorectal cancer [29], non-small-cell lung cancer [30], hepatocellular carcinomas [31], and breast cancer [32]. However, in our study, EIF3H was downregulated, and the relationship between EIF3H and AD and the potential mechanisms and functions remain unknown and need to be further explored. *RAD51C*, a member of the RAD51 encoded family, is located on chromosome 17q22 and mainly participates in the process of homologous recombination and DNA repair [33]. Importantly, a previous study revealed that *RAD51C* mutation may regulate oxidative stress levels and DNA damage characteristics [34]. Interestingly, Lin et al. found that cognitive aging is influenced by the interactions between *EXO1* and *RAD51C* genes [35]. In addition, another study identified seven genes, including *RAD51C*, as potential biomarkers for AD diagnosis [36]. Consistent with the results of our study, Wang et al. found that *RAD51C* is downregulated in AD, which was consistent with our present results [36]. However, due to a few related studies, it remains unclear whether this is related to the formation of amyloid plaques or other pathological mechanisms of AD.

Although little is known about *FAM162A*, it has been shown to act as an HIF-1*α*-responsive proapoptotic molecule, also known as human growth and transformation dependent protein (HGTD-P). As previously confirmed, it may play a role in promoting mitochondrial apoptosis induced by hypoxia [37, 38]. For example, relevant studies demonstrated that *FAM162A* overexpression induces canonical mitochondrial cell death in multiple cells, including prostate cancer cells [37]. Furthermore, Lee et al. found that multiple genes that are upregulated, including *FAM162A*, are cardiac enriched and may have an effect on the progression of heart failure [39]. *FAM162A* was identified as an upregulated gene in several diseases [39, 40]; however, we found that this gene was downregulated in AD, and the specific mechanism involving AD remains to be further explored.

Biliverdin reductase A (BLVRA), an isozyme of biliverdin reductase, plays a pivotal role in maintaining the cellular redox balance. The impairment of BLVRA induced by oxidative stress is responsible for the increased accumulation of A*β* and tumour necrosis factor-alpha, which remarkably results in the onset of brain insulin resistance as the pathology of AD progresses [41]. Interestingly, a previous study found that the reduction and activation of BLVRA protein occur early in the 3xTg-AD mouse brain before uniformly elevating the pathological features of AD [41]. A strong negative correlation was found between BLVRA and BACE1, thereby favouring the direct involvement of BLVRA in the regulation of BACE1.[42] BACE1 is the rate-limiting enzyme in the generation of A*β*. Inhibiting the enzymatic activity of BACE1 could reduce the production and toxicity of A*β*. [43] Additionally, decreased BLVRA levels impaired oxidative stress and neuroprotection, resulting in increased tau phosphorylation in early-stage AD, suggesting that it is a promising therapeutic target for AD [44].

V-type proton ATPase subunit H (ATP6V1H) is a member of the V-type proton ATPase family [45] and is crucial for all types of biological processes, including promoting cellular function and development, regulating protein degradation, and intracellular compartment acidification of eukaryotic cells [46, 47]. Although there is no study indicating a direct relationship between ATP6V1H and AD until now, some studies involving encoded proteins and metabolic processes of BACE revealed that *ATP6V1H* mutations may lead to increased BACE activity [48]. In addition, increased BACE activity may lead to the accumulation of A*β* in the brain, resulting in the formation of amyloid plaques. [49, 50] Considering the lysosome pathway is a vital degradation pathway of the BACE protein, V-ATPase also plays a crucial role in lysosomal acidification [42, 43, 49–52].

In this study, WGCNA and LASSO regression analyses were used to identify targets and molecular pathways related to AD. Moreover, ROC curve verification demonstrated that six key genes showed high performance in predicting specificity and sensitivity. However, except for the lack of in vivo and in vitro experimental verification, the present study has some limitations that must be considered. Another limitation is the lack of focus on the different stages of AD. Mining the hub genes that predict AD in early stages and providing timely drug intervention can prevent the occurrence and development of AD. Moreover, the predictive ability of the six-gene signature should be verified in other databases; unfortunately, on account of clinical data limitations, we could not find a suitable dataset at present. In future studies, we plan to pay close attention to the relevant information in other databases and conduct in vitro studies, while our ongoing studies will focus on exploring the mechanism of action of *EIF3H*, *RAD51C*, *FAM162A*, *BLVRA*, *ATP6V1H*, and *BRAF* in AD.

## 5. Conclusions

Taken together, using WGCNA and comprehensive analyses, this study provides a better understanding of the role of biomarkers *EIF3H*, *RAD51C*, *FAM162A*, *BLVRA*, *ATP6V1H*, and *BRAF* and provides a biological basis for further studies on AD progression.

## Data Availability

The data is public and can be downloaded from the GEO database for free.

## Supplementary Materials

Supplementary 1 Supplementary Table 1: the results of biological processes of GSEA.

Supplementary 2 Supplementary Table 2: the results of GO enrichment analysis.
